# Chronic oral administration of *Passiflora incarnata extract* has no abnormal effects on metabolic and behavioral parameters in mice, except to induce sleep

**DOI:** 10.1186/s42826-019-0034-9

**Published:** 2019-12-30

**Authors:** Gwang-Ho Kim, Sun Shin Yi

**Affiliations:** Department of Biomedical Laboratory Science, College of Medical Sciences, Soonchunhyang University, Asan, Republic of Korea

**Keywords:** Behavioral abnormality, Insomnia, Metabolic abnormality, *Passiflora incarnata*, Sleep-inducer

## Abstract

Although the number of prescriptions and dependence on sleeping pills are increasing, the associations with unexpected abnormal behaviors and metabolic diseases caused by the overuse of sleeping pills are not well understood. In particular, such as abnormal eating-behavior and the occurrence of metabolic disorders caused by psychological unstable states are reported. For this reason, herbal medicine, which has not had such side effects in recent years, is attracting attention as an alternative medicine/food for sleeping inducer. We have used ethanol extracts from *Passiflora incarnata* (PI) to steadily obtain positive effects on sleep and brain microenvironment. However, as mentioned earlier, sleep-inducing efficacy can only be used safely if the behavioral and metabolic abnormalities do not appear.

Thus, in this study, we used Phenomaster equipment to continuously monitor the movement, feeding, water consumption, gas changes, etc. in C57BL/6 mice at a dose of 500 mg/kg/day for 5 consecutive days with PI extract group compared with the control group. Before sacrifice, differences in body composition of mice were also compared. Monitoring of 24 h/5 days through the equipment showed no change in PI-treated group in anything except for significant decrease in blood melatonin levels and activity after PI administration. Taken together, the statistically insignificance of any behavioral and metabolic phenomenon produced by repeated treatment of PI are not only expected to have an accurate sleep effect, but are also free of side effects of the prescribed sleeping pills. This study has given us greater confidence in the safety of the PI extracts we use for sleep-inducer.

## Introduction

Over the past few decades, the use of sleeping pills has increased dramatically as the number of people who have not experienced proper sleep has increased [11]. Sleep disorders could be accompanied by metabolic and unintentional behavioral problems [5, 6, 24, 26, 31]. This is why it is known that sleeping pills are reported to have the above mentioned side effects. Many side effects of prescription sleeping pills have been reported according to the recent studies, which demonstrated that patients who suffering from irregular sleeping have possibility about metabolic disorders in bodies according to the studies [2, 19, 21]. In particularly, overdose and long-term intakes of sleeping pills are shown abnormal behavior during the sleep, and have reported high mortality rate of insomniac patients [19, 21, 29]. However, unexpected adverse effects of these prescription drugs on the human body and mind are not well known and thus the use of newly developed sleep-inducers needs to be very careful as long as potential side effects of the sleep-inducers are observed [19]. Recently, these drugs have presented a critical problem for both physicians and patients, and herbal medicine, including foods has become an alternative to minimize the problems [23, 30, 36]. Our group recently identified major flavonoids in an ethanol extract from *Passiflora incarnata L.* (PI), commonly known as passion flower, that function as sleep aids. A previous study confirmed that the extract induced sleep by single or/and repeated administration to animals. In the current study, we observed the behavioral and metabolic changes caused by repeated oral PI extract administration for 5 days in mice. The safety issues identified in this animal model will be very important factors in the approval by the Korean Food and Drug Affairs (KFDA) during commercialization of this plant-derived sleep inducer. In the present study, we investigated the safety of PI extract as a substitute for sleep medications, by evaluating metabolic and behavioral changes resulting from repeated oral administration.

## Materials and methods

### Preparation of the extract

*Passiflora incarnata* in Korea was selected and supplied by Natural F&P, and the identification number (n°: N9105701) was assigned for further standard use. In addition, extraction was attempted with the extraction multiple of the material and solvents at 2. Passion flower extract was obtained from the leaves and fruit of PI. Extractions were done with 60% aqueous ethanol for 4 h. The aqueous extract was dried with vacuum-evaporation. After vacuum drying, the extract was standardized, using vitexin 0.3% as a reference compound.

### HPLC analysis

Passionflower extract powder (1 g) was dissolved in 50 ml of 50% ethanol for 10 min with sonication. The sample was filtered with a 0.45 um syringe filter. HPLC was performed with an Agilent 1200 system equipped with a model G1312A binary LC pump, an auto sampler, and a diode-array detector. A C-18 (Waters, SunFire C18) column (250 mm × 4.6 mm id and 5 μm particle size) was used. Standards were purchased from Sigma Chemicals.

Chromatographic separations were performed with a mobile phase consisting of 0.1% phosphoric acid prepared in nanopure water (87%) and 100% acetonitrile (13%) for 60 min. The injection volume was 20 μl, the mobile phase flow rate 1 ml min^− 1^, the oven temperature 35 °C, and the detection wavelength 360 nm.

### Experimental mice and PI oral administration

C57BL/6 mice were used purchased from Charles River Japan to investigate whether chronic PI extract administration adversely effected feeding, activity or caused metabolic changes. Total molecular biological, behavioral and metabolic changes were assessed. The mice were separated into control group (vehicle-treated) and PI (*Passiflora incarnata treated) group*, and monitored with Phenomaster®, an automated combined indirect calorimetry system (TSE System GmBH, Bad Homburg, Germany). The animals were administered vehicle (distilled water; dose equivalent to body weight, same as the PI group) or PI 500 (PI 500 mg/kg/day) orally with sonde for 5 days at 17:00, 2 h before lights off. Before the experiment, mice were acclimated for 2 days in a metabolic chamber with food and water, and subsequent oxygen consumption (VO_2_), carbon dioxide production (VCO_2_) and food consumption were measured for 7 days. The respiratory exchange rate (RER; VCO_2_/VO_2_) was calculated using standard in-house software. Energy expenditure (EE) was demonstrated by the equation as EE = 3.815 × 10^− 3^ × VO_2_ + 1.232 × 10^− 3^ × VCO_2_. Body composition (lean tissue, fat, and fluid in live mice on a bench-top platform) was measured following animal phenotyping with Mini-spec LF50 (Bruker Biospins, The Woodlands, TX). This work was technically supported by the Korea Mouse Phenotyping center, Republic of Korea Project (2013M3A9D5072550) of the Ministry of Science, ICT and Future Planning through the National Research Foundation, Republic of Korea.

Mice were housed at room temperature (22 ± 2 °C) with 60% humidity under a 12-h light: dark cycle (light cycle: dark cycle from 07:00 to 19:00). The animals were provided free access to normal chow diet (2018S; Harlan) and water. The handling and care of the animals conformed to guidelines of current international laws and policies (NIH Guide for the Care and Use of Laboratory Animals, NIH Publication No. 85–23, 1985, revised 1996). The Soonchunhyang University Institutional Animal Care and Use Committee (IACUC) approved all experiments and procedures (Approval number: SCH16–0037, August 17th, 2016).

### Tissue processing

The animals were anesthetized with 1 g/kg urethane (Sigma-Aldrich) and perfused transcardially with 0.1 M phosphate Buffer (pH 7.4) to remove as much blood from the body as possible. Before perfusion, blood was collected from the abdominal vein for measurements of serum serotonin and melatonin. After flushing the blood vessels completely, the brains were removed, and the hypothalamus was isolated. The brain tissues were stored at − 80 °C for later processing.

### Western blot

The isolated hippocampus and hypothalamic chunks were homogenized in lysis buffer (iNtRon Biotechnology). Protein concentrations were determined with a BCA kit (iNtRon Biotechnology). Total proteins (20 μg per sample) were loaded into each lane of 12% SDS-PAGE, electrophoresed, and transferred to PVDF membranes (Bio-Rad Laboratories). Following transfer, membranes were blocked with TBST [100 mM Tris-HCl (pH 7.6), 0.8% NaCl and 0.1% Tween-20], containing 10% skim milk (BD Biosciences). These membranes were incubated with the following primary antibodies: rabbit anti-calretinin (1:3000; Swant) and rabbit anti-GAPDH (glyceraldehyde 3-phosphate dehydrogenase, 1:5000; Cell Signaling Technology) at 4 °C overnight. After further washing, membranes were incubated with horseradish peroxidase (HRP)-conjugated anti-rabbit secondary antibodies (Vector). Immunoreactive signals were detected through enhanced chemiluminescence (Abclon) and recorded with the MicroChemi 4.2 system.

## Results

**Fig. 1:**
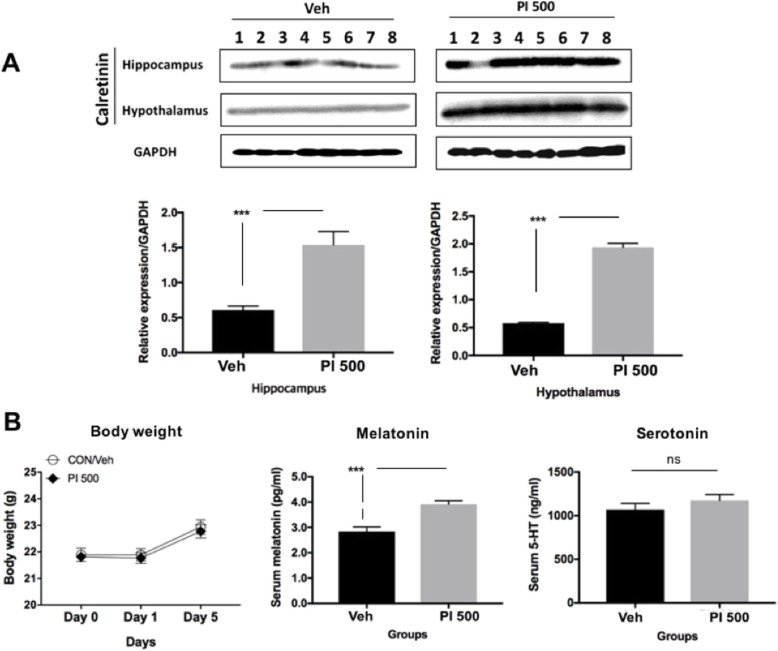
GABA activation and sleep-related hormone levels by repeated oral PI extract administration. **a** Hippocampal and hypothalamic calretinin expressions were analyzed by Western blotting. Significant increases in expression were observed in the PI-treated group, shown by the relative expression of the proteins at the hippocampus and hypothalamus in the control (Veh) and experimental groups (PI 500). **b** Changes in body weight, and changes in sleeping-related hormones in animals after repeated PI extract administration. There was no change in body weight between the two groups. Serum levels of melatonin and serotonin were increased in the PI group. Serum serotonin tended to increase in the PI-treated group, but was not statistically significant, and melatonin showed a significant increase. The error bar represent mean ± standard error (SE). (^***^, *P* < 0.0005; ns, non-significant)

Calretinin expression in the hippocampus and hypothalamus of PI extract (PI 500) administered mice was significantly higher than that of vehicle (Veh)-treated mice (Fig. 1a). Any body weight alterations observed during repeated PI administration were comparable in Veh mice. Serum melatonin of the PI 500 mice showed statistical significance compared to the Veh mice, and the serum serotonin levels were high in the PI group, but were not statistically different from Veh mice (Fig. 1b).

**Fig. 2:**
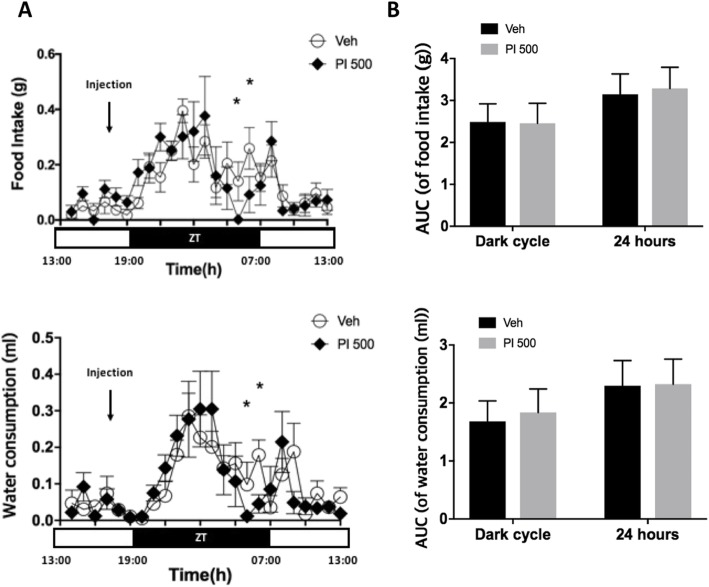
Real-time changes in feed and water intake over 24 h by light cycles between Veh- and PI 500-treated animals. **a** Veh and PI extract (500 mg/kg) were administered 2 h before the lights were turned off. However, there was a significant decrease or declining trend in both feed and water intake in the PI 500 group 2–4 h before the lights were turned on. **b** After the lights were turned off, both the food intake and water consumption were increased in both groups, but no difference in volume between the two was found. (AUC; area under curve). The error bar represents mean ± standard error (SE). ZT stands for zeitgeber time. (^*^, *P* < 0.05; ns, non-significant)

Food intake and water consumption were recorded by Phenomaster metabolic cages for 5 days. All data obtained from the Phenomaster metabolic cages were transformed to provide real-time average consumption for the 24-h light/dark cycles. Food intake and water consumption volumes were not statistically different, except 2 and 3 h before lights-on (Fig. 2a). Total consumption volumes during each dark cycle and over the entire 24 h were not different from each other (Fig. 2b).

**Fig. 3:**
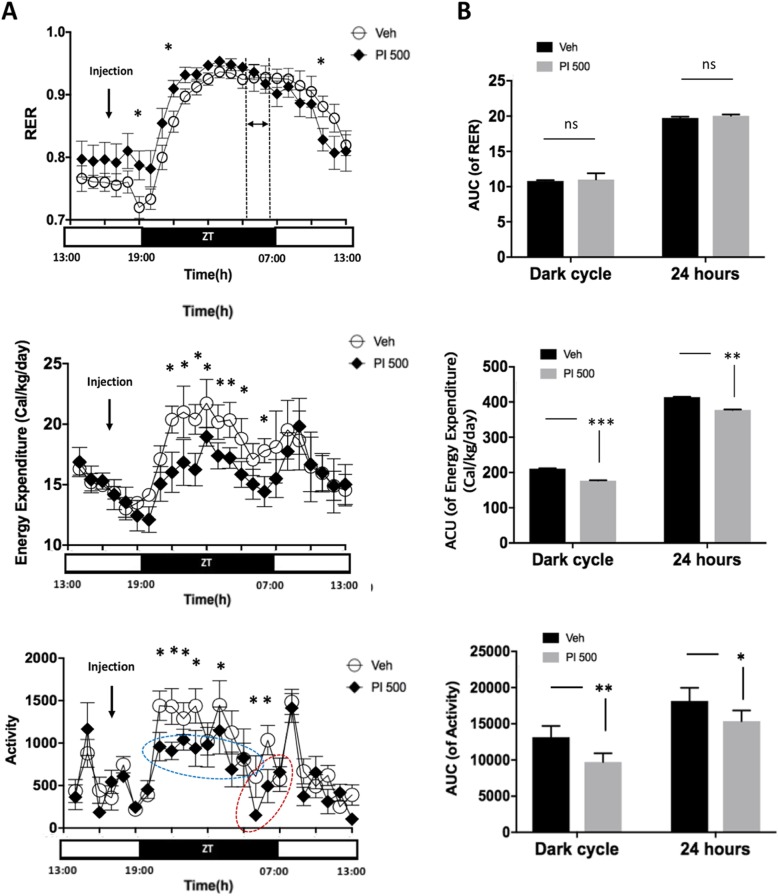
Real-time changes in RER, EE and activity over 24 h by light cycles between Veh- and PI 500-treated animals. **a** Real-time change tracking of RER, EE and activity over 24 h in mice treated with vehicle and PI extract. **b** The area under curve (AUC) demonstrated by bar graphs during dark cycle and 24 h in RER, EE and activity in Veh and PI 500 mice. The vehicle and PI extract were administered orally 2 h before lights- off. EE and activity showed statistical significance between the groups. The error bar represents mean ± standard error (SE). ZT stands for zeitgeber time. (^***^, *P* < 0.0005; ^**^, *P* < 0.005; ^*^, *P* < 0.05; ns, non-significant)

The RER in PI 500 was higher during the light cycle, and was higher about 11 h following PI oral administration (Fig. 3a RER). However, following 04:00~06:00 (duration between dot-lines), the pattern of the RER reversed, and the Veh group RER began to rise.

On the RER real-time graph, only 3 time-points were shown to be significant (Fig. 3a RER), however, the AUC of RER for the dark cycle and the entire 24-h period were not significant (Fig. 3b AUC of RER). Interestingly, EE results were clearly down-regulated in the PI 500 group during dark cycles after PI administration in both EE real-time (Fig. 3a EE) and AUC of EE (Fig. 3b AUC of EE). Activity data were consistent with the above results (Fig. 3a Activity and Fig. 3b AUC of Activity). After PI treatment, the PI 500 group showed lower activity during dark cycles on the real-time activity graph, than the Veh group (Fig. 3a Activity). The PI 500 group maintained low activity during dark cycles and cumulative activity in the PI 500 group was significantly lower (Fig. 3b AUC of Activity).

**Fig. 4:**
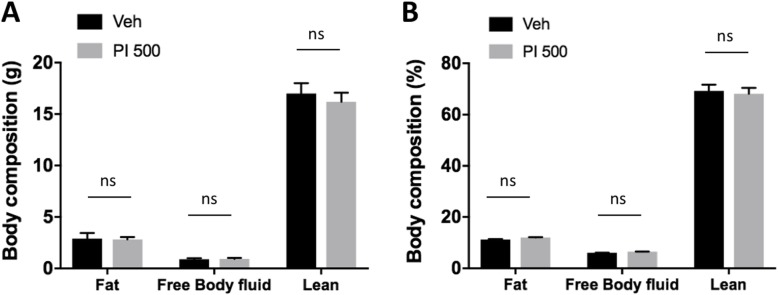
Body composition after repeated oral administration of Veh and PI extract. **a** Body compositions of fat, free body fluid and lean mass (gram). **b** Body composition ratio (%) by fat, free body fluid and lean. There was no significant index change caused by chronic PI administration for 5 days. The error bar represents mean ± standard error (SE) (ns, non-significant)

Just before sacrifice of the animals, body compositions of the live mice were quickly measured (Mini-spec LF50). The obtained fat, body fluid, and lean mass measurements generally are known to account for about 95% of the total body weight. The body composition data were obtained according to the equipment manufacturer’s protocol, and no index value was statistically different between the Veh group and the PI 500 group (Fig. 4a and b). This data showed that repeated PI extract oral administration did not change body composition.

## Discussion

Many studies have shown that the relationship between sleep quality and metabolic rate is very closely associated to their systemic regulatory mechanisms, and data on metabolic rates has been used to predict subjects’ sleep state [6–8, 10, 18, 25, 32, 34, 35]. Although the quality of sleep affects metabolic activity rate, many studied have reported that metabolic rate may affect sleep quality [6, 8, 10, 18, 25, 32, 34]. Therefore, sleep and metabolic rate can play very important roles to each other. Neuropsychiatric abnormal behaviors and symptoms, such as depression, anxiety and suicide have been reported in numerous studies [1, 13, 17, 38]. Insomnia itself is an adverse event, but very dangerous physical and mental changes caused by sleeping pills have also been reported [2, 4, 15, 16, 19, 25, 29, 33]. Drinking tea made from passion flowers, particularly *Passiflora incarnata*, has long been known to provide stability and induce sleep [22]. It is classified as a very safe herb, registered as foods with the Korea Ministry of Food and Drug Safety (KFDA). Therefore, we standardized the process of PI extraction with ethyl alcohol to make a safe sleep-inducing product in the present study (Additional file 1), and confirmed the side effects of certain changes in the metabolic rate, which also occur with prescription sleeping pills. Vitexin was believed as a main product in our established extract method. Many studies have reported that vitexin has the ability to induce sleepiness, is anti-diabetic, anti-inflammatory, and effective for sleep improvement [9, 14, 20]. PI extracts showed increased calretinin at the hippocampus and hypothalamus known as calcium binding protein secreted by GABAergic neurons [12, 27, 37]. Parvalbumin is also known to a marker of GABAergic neurons [3, 39], and parvalbumin protein expression was higher in the hippocampus of PI administered animals than in non-treated animals in our previous study (data not shown), and was accompanied by a significant increase in blood melatonin levels (Fig. 1). Serotonin, a kind of catecholamine, plays a very important role as a wake-cycler [3, 28], and is a major regulator of melatonin secretion [28]. However, although serotonin stimulates the secretion of melatonin, it also plays a role in awakening sleep, so very careful interpretation is needed. In the present study, the PI-treated group showed a tendency to serotonin increased, but it was not significant (Fig. 1). Since blood sampling time was 2 h before the lights went out, serotonin levels were lowered, and melatonin stimulation was increased instead. Increased melatonin has been suggested to enhance immune responses by decline in the levels of superoxide anion radical produced by heterophils [28].

There was no difference in the volumes of feed intake or amount of water consumption observed in mice repeatedly administered Veh or PI for 5 days (Fig. 2b), however, there was a significant difference in one time period (Fig. 2a). The intake of feed and drinking water decreased in the PI-treated group from about 2 h before lights were turned on, and the tendencies of feed and water alterations were very similar in the graphs (Food intake and Water consumption in Fig. 2a). The mean real-time graph of 24 h (× 5 day) after administration of vehicle and PI extract shows that the RER (VCO_2_/VO_2_) value remained high for about 9 h after the lights off, and the graph crossed about 2~3 h before the lights on (Fig. 3a). However, the accumulated RER values (AUC of RER) were significant for the dark cycle and 24 h. In general, it is known that energy expenditure (EE) is about 15% less in the sleeping state than the awake state [34]. Our data showed that EE of the PI 500 group decreased by about 16% for 12 h (dark cycle) after the administration of PI extract (Fig. 3a EE). However, the total EE (AUC of EE) result for 24 h showed an 8.8% difference between the groups (Fig. 3b AUC of EE). The PI 500 group also showed statistical significance for total AUC of EE (Figs. 3b). The activity was also significantly decreased after lights-out following the PI administration (^*^; *P* < 0.05; * marks in the blue dot-circle and the red dot-circle). The activity increased gradually from 2 h (in the red dot-circle) before the lights were turned on, and activity after lights-on was similar to that of the control group. The total activity (AUC of activity) was also significant (^**^, *P* < 0.005).

The alterations of body composition between Veh- and PI-treated groups did not show any significant differences (Fig. 4a and b). This result implies that repeated administration of PI extract did not affect any body composition factors due to metabolic changes (Fig. 4).

## Conclusion

These results indicate that PI treatment was effective in increasing GABAergic neuron activity and blood melatonin levels, evidenced by a significant decrease of EE observed at the time when mice are generally active in the dark cycle. In other words, no increase in appetite or increase in body weight was observed, and any body compositions were not changed; only sleeping was changed. Since there have been reports of behavioral abnormalities and metabolic changes that may be caused by the repeated use of diverse prescribing sleeping pills, we tried to find out whether the repeated administration of PI extract may cause such problems in the animal models. Taken together, we did not find any side effects of abnormal metabolic phenotypes or behaviors, such as hyperphagia or unexpected metabolic changes by repeated administration of PI extract to mice for 5 days (Additional file 2). We confirmed the use of PI extract showed only sleep-inducing effects, at least in animal models, without causing any adverse behavioral or metabolic disorders through this study.

## Supplementary information

**Additional file 1.** HPLC chromatograms obtained from extract of passion flower. Peak 1, isoorientin; peak 2, orientin; peak 3, vitexin; peak 4, isovitexin

**Additional file 2.** Conceptual diagram of graphic research results. No abnormalities were found in the PI extract for the various side effects of sleep-inducing substances

## Data Availability

There was some supporting data available for this work. The datasets used and/or analyzed in this study are available from the corresponding author on reasonable request.
