# Supplementary figures and images for: Chronic oral administration of Passiflora incarnata extract has no abnormal effects on metabolic and behavioral parameters in mice, except to induce sleep
Source: Lab Anim Res. 2019 Dec 30;35:31. doi: 10.1186/s42826-019-0034-9 (PMC7081546; doi:10.1186/s42826-019-0034-9)

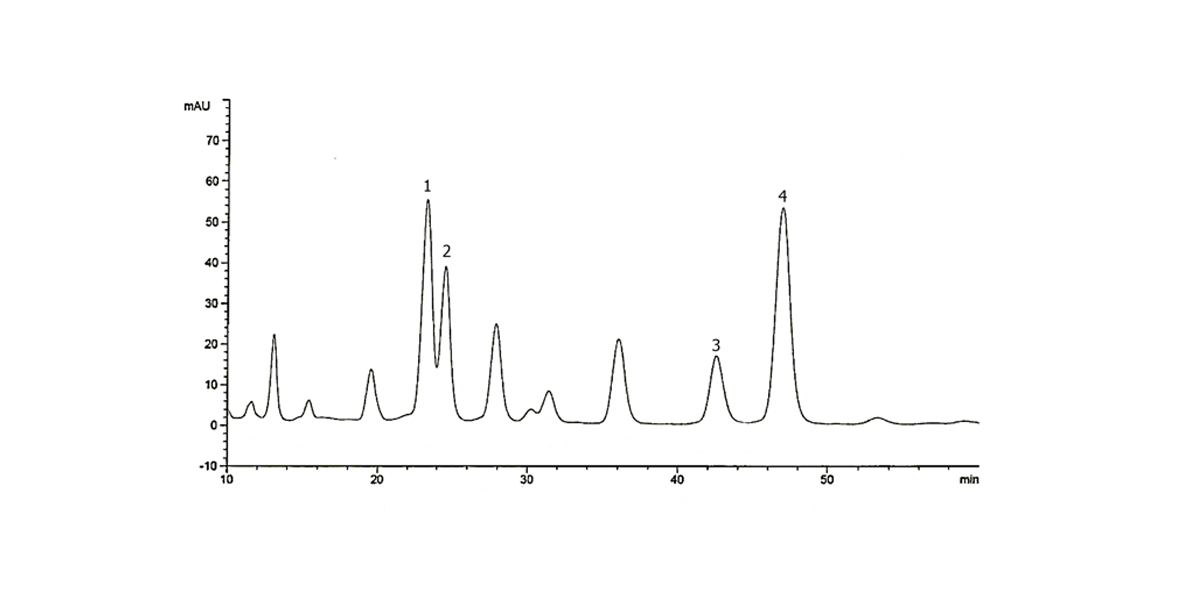

Supplement: Supplementary file 1 — Additional file 1. HPLC chromatograms obtained from extract of passion flower. Peak 1, isoorientin; peak 2, orientin; peak 3, vitexin; peak 4, isovitexin [file 42826_2019_34_MOESM1_ESM.jpg]

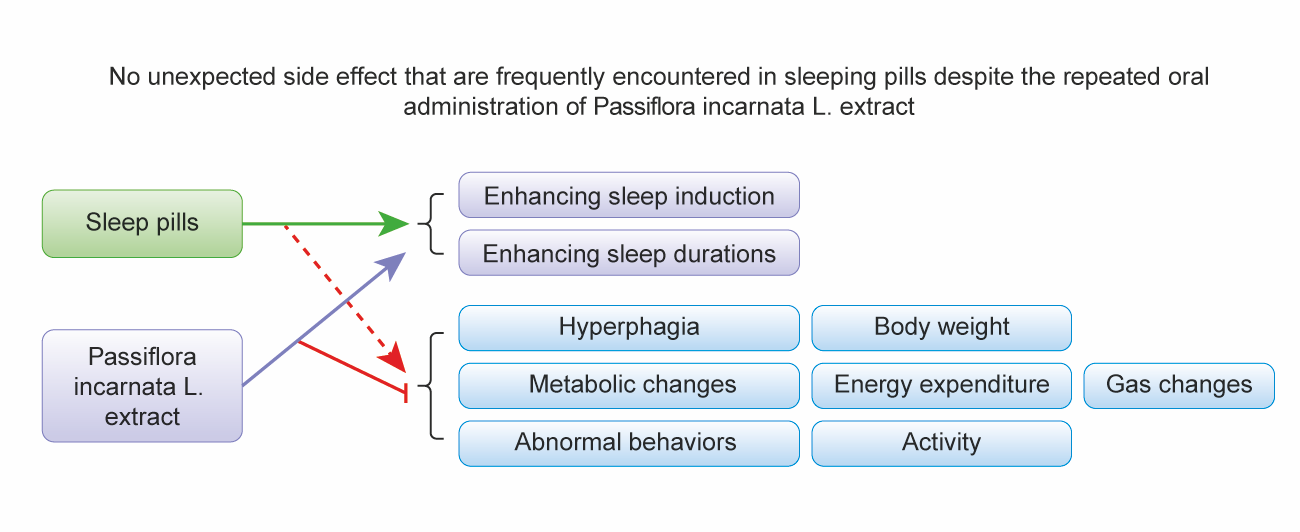

Supplement: Supplementary file 2 — Additional file 2. Conceptual diagram of graphic research results. No abnormalities were found in the PI extract for the various side effects of sleep-inducing substances [file 42826_2019_34_MOESM2_ESM.tif]
